# Dormancy-release and germination improvement of Korean bellflower (*Campanula takesimana* Nakai), a rare and endemic plant native to the Korean peninsula

**DOI:** 10.1371/journal.pone.0292280

**Published:** 2023-10-17

**Authors:** Hyeon Min Kim, Jun Hyeok Kim, Jae Hyeon Lee, Gun Mo Kim, Mi Hyun Lee, Chung Youl Park, Do Hyun Kim, Da Hyun Lee, Kyeong Min Kim, Chae Sun Na

**Affiliations:** Division of Wild Plant and Seeds, Baekdudaegan National Arboretum, Bonghwa, Republic of Korea; Nuclear Science and Technology Research Institute, ISLAMIC REPUBLIC OF IRAN

## Abstract

Korean bellflower (*Campanula takesimana* Nakai) is a rare and perennial herb with medicinal and ornamental values, is endemic to the Ulleung Island of Korea. In this study, we investigated the dormancy-release and germination characteristics of *C*. *takesimana* (Campanulaceae) seeds by subjecting them to varying temperatures (5, 10, 15, 20, and 25°C and diurnal/nocturnal temperatures of 15/6, 20/10, and 25/15°C), cold stratification periods (0, 4, 8, or 12 weeks at 5°C), and gibberellic acid (GA_3_) concentrations (0, 10, 100, or 1,000 mg·L^-1^ at 15/6°C and 25/15°C) to identify the ideal seed propagation conditions. The seeds were stimulated to germinate (at 25°C, 12-h photoperiod with fluorescent lamps at 40 ± 10 μmol∙m^-2^∙s^-1^) after cold stratification. To examine the germination characteristics, the seeds were tested for water imbibition and found to readily absorb water. The seeds exhibited underdeveloped embryos during dispersal, showed final germination of 37.00% ± 4.43 at 25°C and were not influenced by temperature. The seeds subjected to 0, 4, 8, or 12 weeks of cold stratification germinated at a success rate of 22.00% ± 4.76, 87.00% ± 6.80, 79.00% ± 2.52, and 77.00% ± 1.91, respectively. Additionally, the germination characteristics, which were based on final germination, mean germination time, and germination velocity (Timson index), were significantly greater in the seeds pretreated with 1,000 mg·L^-1^ GA_3_ at 25/15°C than in seeds pretreated with 0 mg·L^-1^ GA_3_. Overall, the seeds broke dormancy with GA_3_ and short-term cold stratification. Therefore, we concluded that *C*. *takesimana* seeds have non-deep, simple, morphophysiological dormancy, and pretreatment with cold stratification and GA_3_ is required for effective seed propagation.

## 1. Introduction

The genus *Campanula* contains approximately 600 species and belongs to the family Campanulaceae (bellflower family), which is distributed mainly in the subtropical and mountainous habitats of the Northern Hemisphere [1]. The genus *Campanula* has approximately three species that are distributed throughout Korea; among them, *Campanula takesimana* (English name: Korean bellflower) is rare (least-concern, LC) and endemic to Ulleung Island, which is located in the East Sea of Korea [2] (see also KNA 2022). *C*. *takesimana* is a striking halotolerant, herbaceous, and perennial species and grows to 30–100 cm. The species has light pink or tinged-peachblow petals, blooms in July–August, and grows favorably in both well-drained and dry soils. In addition, *C*. *takesimana* is one of the most popular ornamental plants and an important resource for gardening and landscaping owing to its long flowering period and high ornamental value [3, 4]. Traditionally, the stems, leaves, and flowers of *C*. *takesimana* have long been used as an ethnobotanical and/or medicinal plant in Korea [2, 5]. The antioxidative effect of *C*. *takesimana* is revealed by the DPPH and ABTS radical scavenging ability, reducing ability, and SOD-like activity of its ethanol extract and fractions; therefore, it is considered to be a natural material for food additives and cosmetics [6]. This important species needs to be investigated using not only in situ measures but also ex situ conservation measures through the continuous monitoring and proliferation of its habitats.

The population of wild plants is decreasing recently due to climate change, forest burning, and native habitat destruction, and basic data on germination characteristics of seeds are required for forest restoration and conservation of these native plants. Moreover, a mass propagation method must be established to industrialize plant materials; therefore; it is important to create a propagation protocol [7]. Appropriate methods that should be considered for the propagation of plants based on plant species, habitat, life cycle, and characteristics include asexual reproduction, sexual reproduction, and tissue culture. For sexual reproduction, a large number of individuals can be secured sufficiently, and it does not require special equipment, labor, time, and facilities. In addition, sexual reproduction increases genetic diversity by mixing the genes of male and female plants. However, it is difficult to secure a large number of plants if seed dormancy cannot be broken. Seed dormancy is the self-suppression of germination under appropriate environmental conditions, such as temperature, light, soil type, water regimes, and climate changes, and it delays the emergence of seedlings over time [8–10]. Although seed dormancy is a critical plant trait from ecological and conservation perspectives, the understanding of how to overcome it could enable the use of wild plant species that are potentially valuable to ornamental horticultural and gardening industries [11, 12]. From the perspective of forest restoration, it is necessary to not only understand the ecology of wild plants but also understand the propagation method of seeds with high genetic diversity for restoration [13]. Thus, regardless of seed source, species-specific knowledge of dormancy-release and germination requirements is needed for the successful cultivation of plants from seeds for application in different industries [14].

Seed dormancy is classified based on the developmental status of the embryo, water absorption capacity, and interrelationships of external/internal phytohormones in the seeds. Worldwide, 50–90% of wild plants produce mature seeds that are dormant and show different types and levels of dormancy [15]. Seed dormancy is broadly classified into five types: physiological dormancy (PD), morphological dormancy (MD), morphophysiological dormancy (MPD), physical dormancy (PY), and combinational dormancy (PY + PD) [15–17]. In the family Campanulaceae, which includes the *Campanula* genus, seeds of several species do not germinate immediately because the embryo is underdeveloped at the time of seed dispersal. Baskin et al. [13] reported that the underdeveloped embryo in seeds of montane species of Campanulaceae from Hawaii increased from 87 to 179% before the radicle protruded from the seed, depending on the species. Consequently, MD and/or MPD types frequently occur in the Campanulaceae family because of underdeveloped embryos, but some species of the same family have seeds with fully developed embryos and either PD or non-dormancy (ND). Seeds with MD only need time to grow fully developed embryos to a critical length before radicle protrusion and do not require pretreatments for dormancy-release [18]. However, in MPD, in which PD and MD are combined, the seeds require more time for embryos to grow to a critical size, and pretreatments, such as the use of cold (0–10°C) and/or warm (≥15°C) stratification, gibberellins (GAs), and ripening in dry storage, are needed to release the dormancy [16, 19]. MPD has been classified into nine levels, and systematic studies have developed classification systems to identify the exact kind of dormancy in seeds [16]. Phytohormones are widely used during dormancy-break and germination of seeds. Among them, exogenous GAs such as GA_3_, GA_4_, GA_7_, and GA _4+7_, have been used to overcome PD or MPD, enhance germination, promote embryo growth, and stimulate radicle emergence in many plant species [20–26]. However, GAs does not enhance germination in all plant species, and seed germination may be inhibited depending on the method, immersion time, and/or concentration of GA solution used. Thus, the ideal concentration of GAs required to break dormancy should be investigated before they are used as a pretreatment.

Based on previous studies of seeds of species of Campanulaceae, we hypothesized that *C*. *takesimana* seeds would have one of the four dormant classes: ND, MD, PD, and MPD [13, 14, 27–29]. To test our hypothesis, we investigated the (1) constant and fluctuating temperature regimes required to improve germination percentage, (2) morphology of embryo growth and seed germination under laboratory conditions, and (3) effects of cold stratification and GA_3_ on dormancy-release. Our findings provide a reference for species conservation through mass propagation protocols and can aid horticulturalists and seed ecologists in efficiently producing *C*. *takesimana* seedlings.

## 2. Materials and methods

### 2.1 Plant material

Mature *C*. *takesimana* seeds were collected on September 6, 2021, from plants growing in Baekdudaegan National Arboretum, Bonghwa, Gyeongsangbuk-do, Korea. The seeds were cleaned and examined to determine their characteristics, including seed length (*n* = 10), width (*n* = 10), and 1000 seed weight (*n* = 100). Each parameter was repeated four times, and the weight of 1000 seeds was measured using an electronic balance (ML204/01, Mettle Toledo, Columbus, OH, USA). After drying the seeds in a drying room (15°C, relative humidity (RH) of 15%) for one week, they were sealed in a plastic bag and stored at 4°C until the start of the experiment on September 16, 2021.

### 2.2 Water imbibition test

To test for the presence (or not) of PY, seed imbibition of water was determined under laboratory conditions (approximately 23 ± 2°C, RH of 40–50%). Four replicates of 100 *C*. *takesimana* seeds were weighed using an electronic balance (ML204/01, Mettler Toledo, Columbus, OH, USA), and then seeds were placed on two layers. Thereafter, *C*. *takesimana* seeds from each replicate were individually placed on two layers of filter paper (Whatman No. 2, Toyo Roshi Kaisha, Ltd., Tokyo, Japan) moistened with distilled water in four 90 × 15 mm plastic Petri dishes (SPL Life Sciences Co., Ltd., Pocheon, Korea). After 3, 6, 9, 12, 24, 72 and 96 h of incubation, each replicate of seeds was weighed. Water was removed from the seed surface with paper towels, and after weighing seeds were returned to the moist filter papers. The percentage increase in mass due to absorption of water by the seeds was calculated using the following formula [16].

$$\%W_{s}=[(W_{i}‐W_{d}/W_{d})]\times 100$$

where W_s_ indicates the increase in seed mass, W_i_ is the seed weight after a given imbibition interval, and W_d_ is the original seed weight before water absorption.

### 2.3 Seed morphology and embryo development

Seeds were cut along the major or minor axis using a razor blade (stainless blade, Dorco, Seoul, Korea). The cross-section was measured and photographed under a digital microscope (DVM6, Leica Microsystems GmbH, Wetzlar, Germany); seed morphology was observed to identify the MD in the seeds. The increase in embryo length in freshly-matured seeds and in seeds just before germination was measured. The ratio of embryo length to seed length (E:S ratio, %) was calculated using the formula [30]. A Scanning Electron Microscope (CX-200; COXEM, Daejeon, Korea) was used to capture the images of the seed coat surface.

### 2.4 Effect of temperature on germination

To identify the optimal temperature for seed germination, seeds were incubated at 5, 10, 15, 20, 25, 15/6, 20/10, and 25/15°C (day/night) for seven weeks in a growth chamber (TGC-130H, Espec Mic Corp., Aichi, Japan). The seeds received light from fluorescent lamps with 40 ± 10 μmol·m^−2^·s^−1^ photosynthetic photon flux density (PPFD) for a period of 12/12 h day/night. Four replicates of 25 seeds were placed in plastic Petri dishes (60 × 15 mm, SPL Life Sciences Co., Ltd., Pocheon, Korea) on a 1% agar (Agar, Sigma-Aldrich, St. Louis, MO, USA) medium, which were sealed with a parafilm (PM-996, Bemis Company Inc., Neenah, WI, USA) during incubation.

### 2.5 Cold stratification period

To examine the impact of cold stratification on dormancy-release for germination, the seeds were incubated for a period of 0, 4, 8, or 12 weeks at 5°C. For each period of cold stratification, four replicates containing 25 seeds were placed in plastic Petri dishes (60 × 15 mm) on a 1% agar medium, which was sealed with a parafilm during incubation. After each cold stratification period for 0, 4, 8, or 12 weeks, four replicates of seeds were transferred to a growth chamber (25°C with a photoperiod of 12/12 h of day/night).

### 2.6 Promotion of germination with GA_3_ concentration

Four concentrations of GA_3_ (≥ 90%, Sigma-Aldrich, St. Louis, MO, USA) solution (0 (control), 10, 100, or 1000 mg·L^−1^) were used to investigate the effects of GA_3_ on the release of dormancy. The control group was soaked in distilled water for 24 h. The seeds were soaked in the four different solutions for 24 h under laboratory conditions (approximately 23 ± 2°C, RH of 40–50%). After which, they were pretreated with GA_3_ and rinsed thrice with distilled water. After then, the seeds were incubated at 15/6°C and 25/15°C in a growth chamber for 12/12 h of day/night. Four replicates containing 25 seeds were placed separately in plastic Petri dishes (60 × 15 mm) on a 1% agar medium, which were sealed with a parafilm during incubation.

### 2.7 Data collection and germination assay

Percent germination (embryo protrusion from seeds) was estimated at 2-day intervals for 49 days. Each seed was considered to have germinated when the radicle emergence reached a minimum of 2 mm. To evaluate the germination process of *C*. *takesimana* seeds, biological parameters such as germination (G) and mean germination time (MGT), were calculated as follows [31]:

$$\%G=G_{49}/N\times 100$$

MGT(days)=∑(T×S)/∑S

where N is the total number of seeds, T is the time in days from day 1 to the final day of the germination test, S is the total number of germinated seeds on day T, and G_49_ is the total number of seeds germinated on day 49 after sowing.

The rate of germination was estimated using a modified Timson index of germination velocity as follows [32]:

$$Timson\;index\;(\%day^{‐1})=\sum (G/t)$$

where G is the total percentage of seeds germinated every 2 days, and t is the total germination period (49 days). A high value of the Timson index indicates a rapid germination rate.

### 2.8 Statistical analysis

All data on germination characteristics were statistically analyzed using the SAS 9.4 software (version 9.4; SAS Institute Inc., Cary, NC, USA). The differences between the embryo-to-seed length ratio (E:S ratio) of seeds at dispersal and just before germination were determined using paired t-tests. The germination experiment results were subjected to analysis of variance and Tukey’s honestly significant difference (HSD) post-hoc test (*p* ≤ 0.05). Regression and graphing were performed using SigmaPlot 12.0 (Systat Software Inc., San Jose, CA, USA).

## 3. Results

### 3.1 Basic characteristics of seeds

The initial mean (± SE) length and width of *C*. *takesimana* seeds were 1.115 ± 0.02 mm and 0.309 ± 0.03 mm, respectively. Additionally, the 1000 seed weight of *C*. *takesimana* was 0.07025 ± 0.001 g.

### 3.2 Water imbibition test

**Fig 1:**
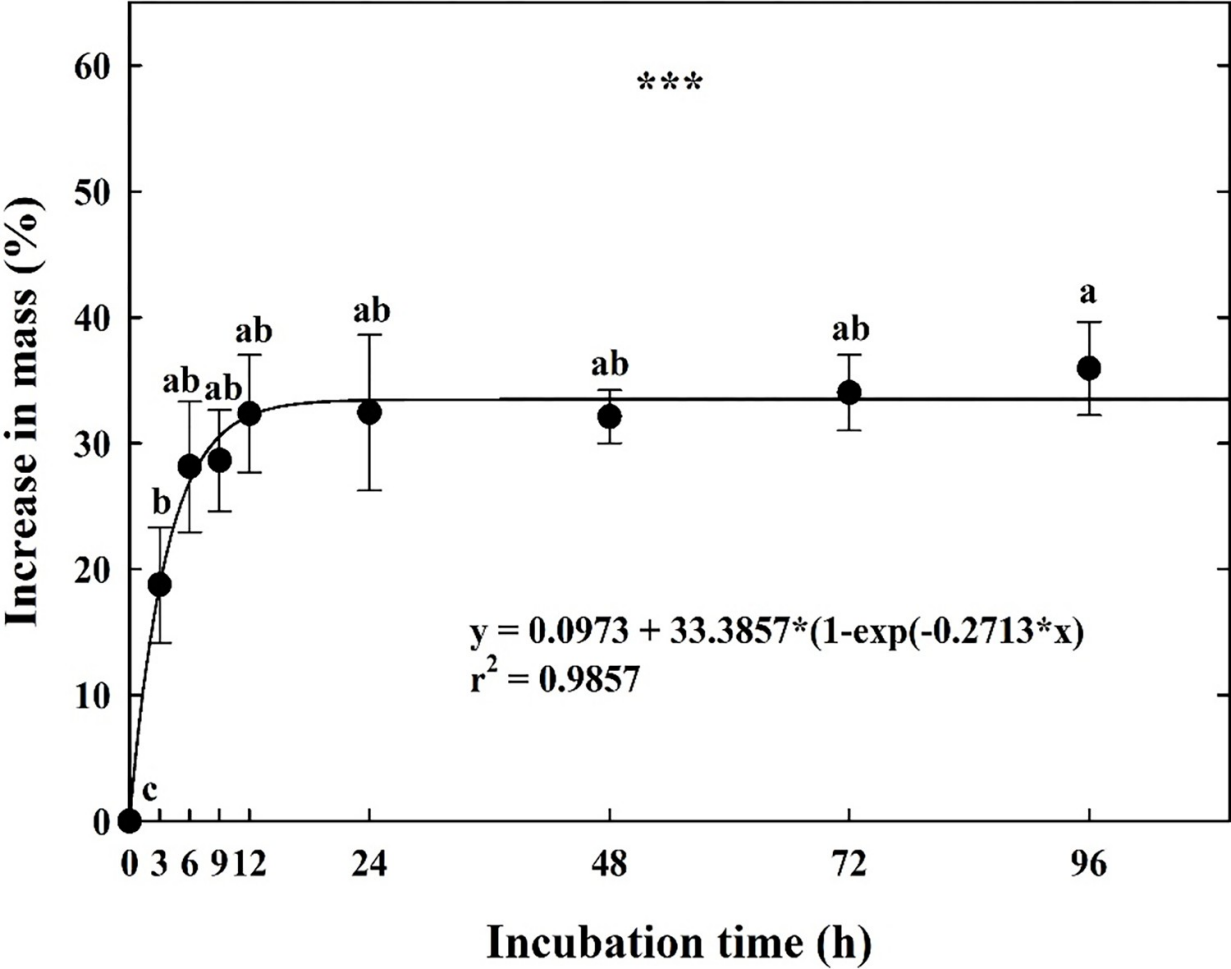
Percentage increase in mass of *Campanula takesimana* seeds during water incubation. The seeds were incubated at ambient temperature (approximately 23 ± 2°C) on filter paper moistened with distilled water for 96 h. The vertical bars represent the standard deviation from the mean (*n* = 4). Different letters in the same column indicate significant differences based on Tukey’s HSD test (*p* ≤ 0.05). *** means significant at *p* ≤ 0.001.

*C*. *takesimana* seeds rapidly imbibed water, and the seed mass increased by 28.13% ± 5.19 and 32.35% ± 4.67 after 6 and 12 h, respectively, of imbibition compared to the initial dry mass (Fig 1). Furthermore, water imbibition peaked at 34.02% ± 3.01 and 35.94% ± 3.71 after 72 h and 96 h, respectively, and there was no further increase in seed mass.

### 3.3 Seed morphology and embryo development

**Fig 2:**
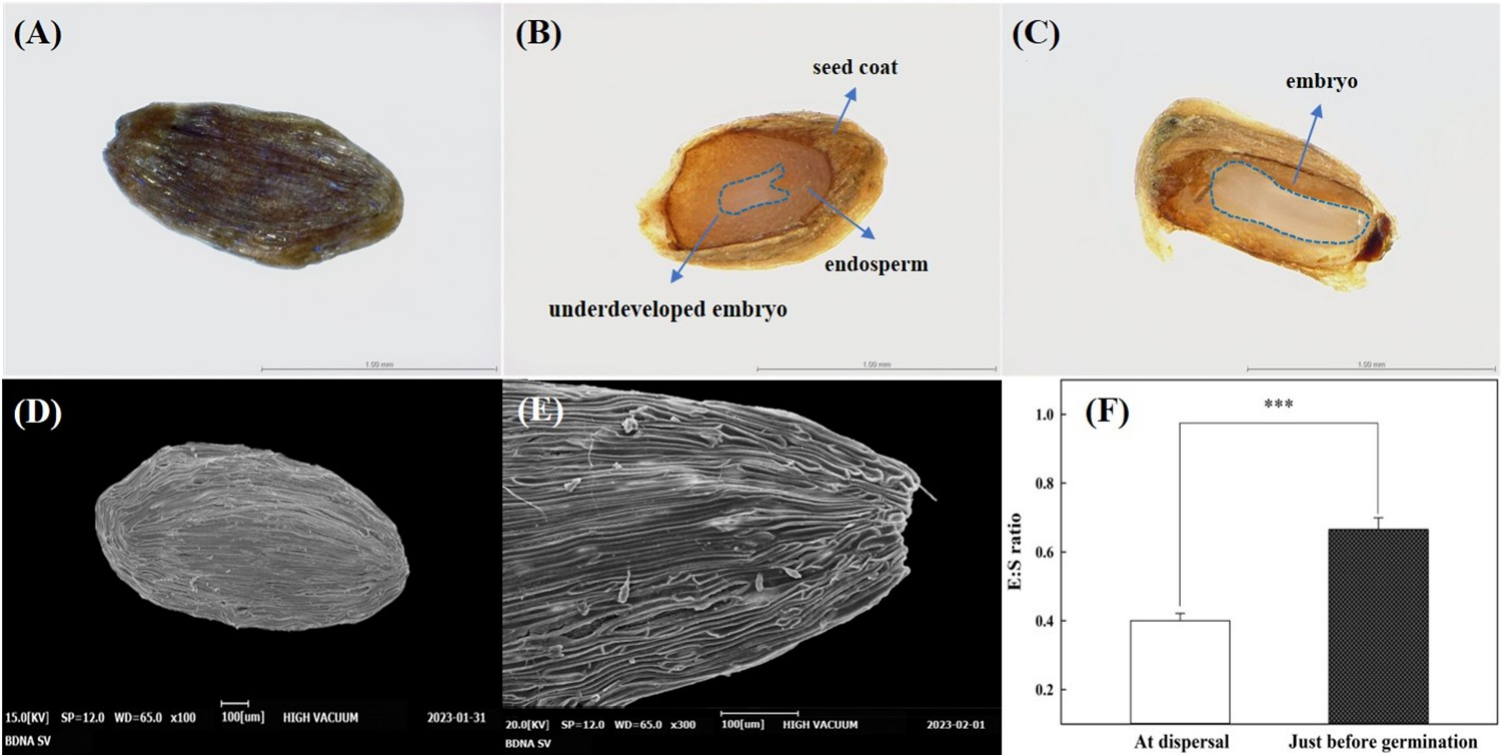
Morphology and anatomy of *Campanula takesimana* seed and embryo. (A) Seed surface, (B) longitudinal section of the underdeveloped embryo at the dispersal stage, (C) longitudinal section of the fully developed embryo at the germination stage, (D-E) seed photographed using a scanning electron microscope (SEM), and (F) Embryo/seed (E:S) ratio in the seeds of *Campanula takesimana* at seed dispersal and just before germination stages. The vertical bars represent the standard deviation from the mean (*n* = 10). Each E:S ratio at dispersal and just before germination stages was compared using a paired *t*-test. *** Significant at *p* ≤ 0.001.

The morphology of the seed surface was flat and round (Fig 2A), and the seed coat had a grain-shriveling structure on its surface in all directions (Fig 2D and 2E). At dispersal, seeds had an underdeveloped embryo (Fig 2B), which became fully developed in the seed germination stage (Fig 2C). The initial mean (± SE) lengths of the seed and embryo were 1.16 ± 0.03 mm and 0.47 ± 0.03 mm, respectively. Just before germination, the lengths of seed and embryo were 0.94 ± 0.04 mm and 0.62 ± 0.05 mm, respectively. The E:S ratio of *C*. *takesimana* seeds at the time of dispersal was 0.40 ± 0.02 but was 0.67 ± 0.03 just before germination, indicating a significant increase of 67.50% during germination (Fig 2F).

### 3.4 Effect of temperature on germination

**Fig 3:**
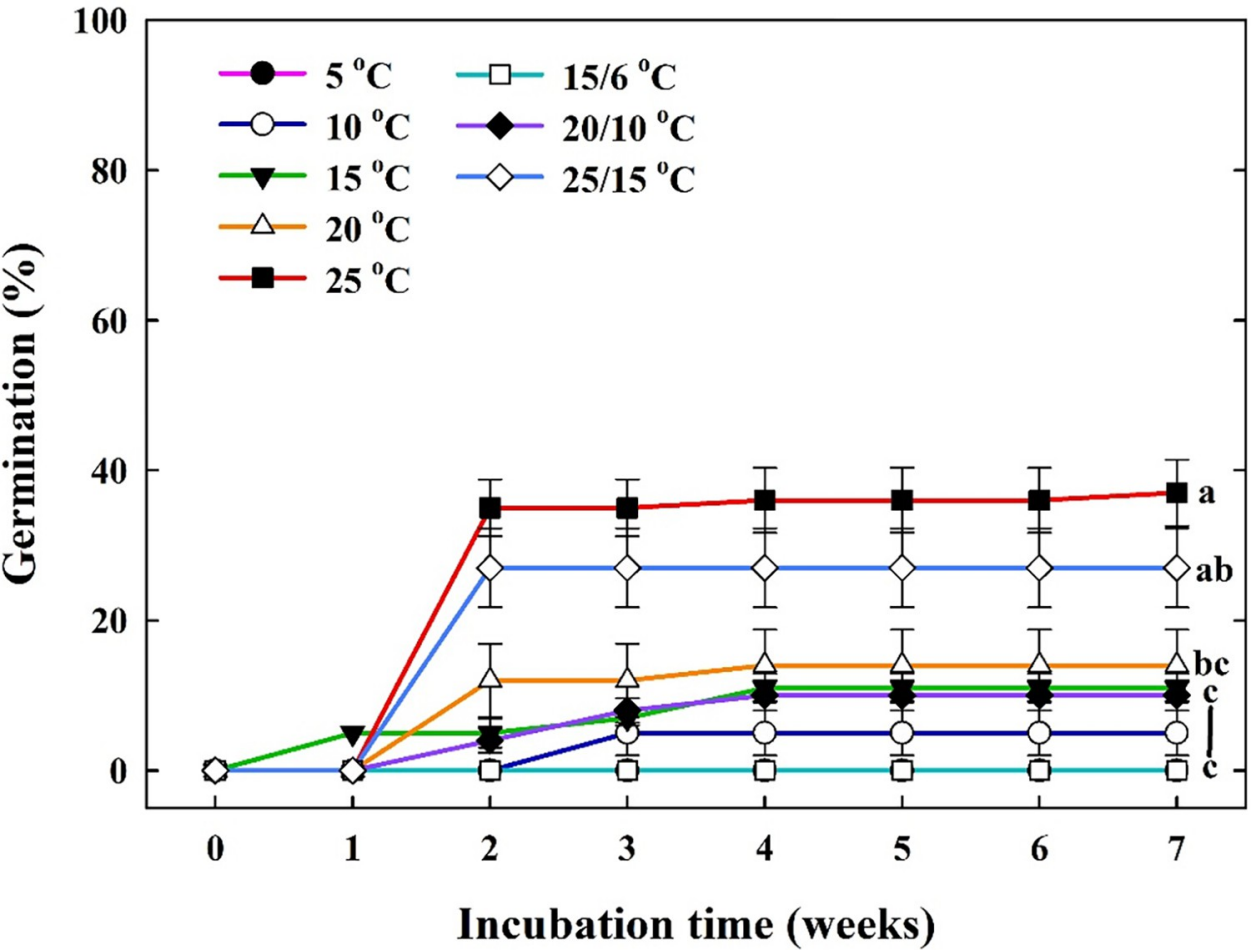
Effects of constant temperature (5, 10, 15, 20, and 25°C) and fluctuating temperature regimes (15/6, 20/10, and 25/15°C) on the cumulative germination of *Campanula takesimana* seeds after 7 weeks of incubation. The vertical bars represent the standard deviation from the mean (*n* = 4). Different letters in the same column indicate significant differences based on Tukey’s HSD test (*p* ≤ 0.05).

Most of the *C*. *takesimana* seeds started to germinate between 1 and 2 weeks and showed a greater tendency to germinate at higher (25°C and 25/15°C) than at lower temperatures (Fig 3). However, there was little or not increase in germination percentages at any of the temperature regime after 3 weeks. Although MGT values tended to decrease as the incubation temperature increased, there was no significant difference in the values in the temperature regime, except at 5, 10, and 15/6°C (Table 1). The relatively high temperature (25°C) significantly increased the germination velocity for TGI (28.58% ± 3.29 day^-1^) compared to the other temperature regimes.

**Table 1:** Effects of constant temperature (5, 10, 15, 20, and 25) and fluctuating temperature regimes (15/6, 20/10, and 25/15°C) on the germination characteristics of *Campanula takesimana* seeds under seven weeks of incubation.

Temperature (°C)	Germination (%)	MGT (day)	TGI (% day^-1^)
**5**	0.00 ± 0.00 c	50.00 ± 0.00 a	0.00 ± 0.00 c
**10**	5.00 ± 3.00 c	36.00 ± 8.08 a	2.90 ± 1.74 c
**15**	11.00 ± 1.91 c	15.79 ± 1.78 b	7.60 ± 1.10 c
**20**	14.00 ± 4.76 bc	15.41 ± 3.24 b	10.56 ± 3.81 bc
**25**	37.00 ± 4.43 a	12.24 ± 0.87 b	28.58 ± 3.29 a
**15/6**	0.00 ± 0.00 c	50.00 ± 0.00 a	0.00 ± 0.00 c
**20/10**	10.00 ± 2.00 c	16.31 ± 1.50 b	6.90 ± 1.32 c
**25/15**	27.00 ± 5.26 ab	11.28 ± 0.14 b	21.42 ± 4.14 ab
**Significance**	***	***	***

MGT, Mean germination time; TGI, Timson germination index.

^a,b,c^Mean separation within columns by Tukey’s HSD test (*p* ≤ 0.05).

*** Significant at *p* ≤ 0.001.

### 3.5 Cold stratification period

**Fig 4:**
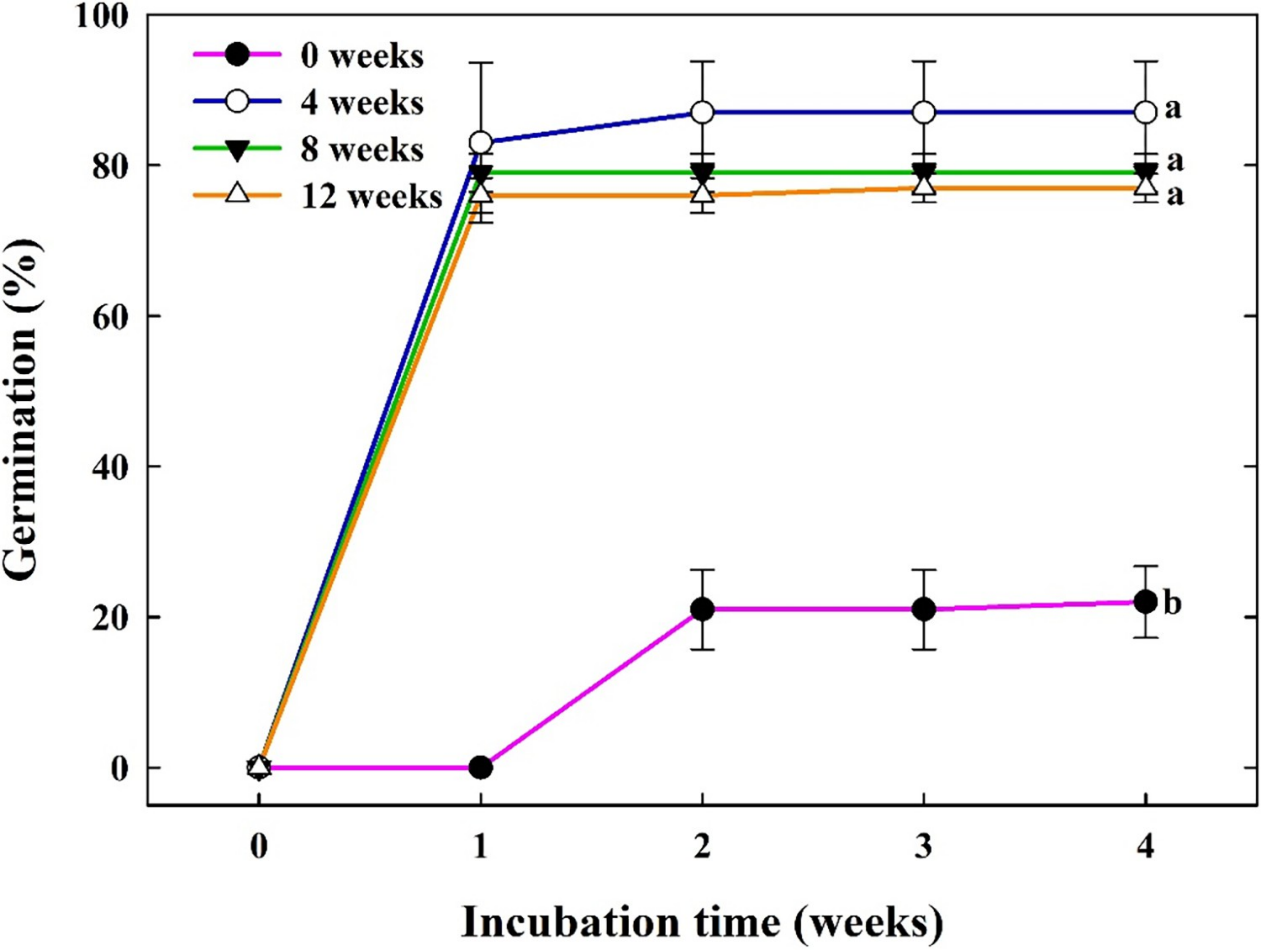
Effects of cold stratification pretreatment (0, 4, 8, or 12 weeks at 5°C) on the cumulative germination of *Campanula takesimana* seeds under 4 weeks of incubation. Seeds were incubated at 25°C after the stratification treatment. The vertical bars represent the standard deviation from the mean (*n* = 4). Different letters in the same column indicate significant differences based on Tukey’s HSD test (*p* ≤ 0.05).

The average germination of control seeds without cold stratification (0 weeks) during the 4-week incubation period was less than 22.00% ± 4.76 (Fig 4). However, after all periods of cold stratification seeds incubated at 25°C for 4 weeks germinated to 77.00 to 87.00%.

### 3.6 Promotion of germination with GA_3_ concentration

**Fig 5:**
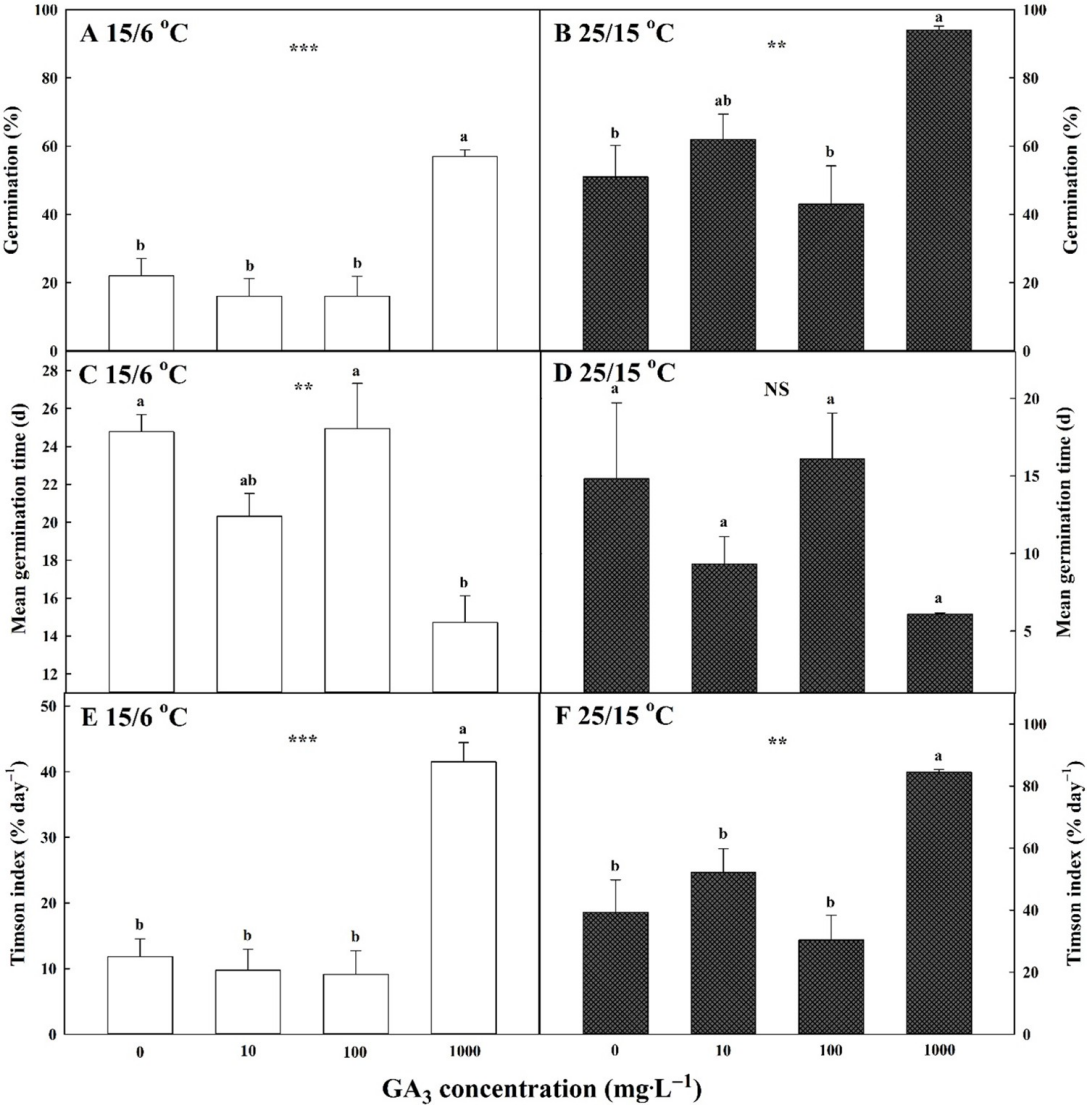
Effects of GA_3_ pretreatment (0, 10, 100, or 1000 mg·L^-1^) and two temperature regimes (15/6 and 25/15°C) on germination (A, B); mean germination time (C, D); and Timson index (E, F) of *Campanula takesimana* seeds under seven weeks of incubation. Vertical bars represent the standard deviation from the mean (*n* = 4). Different letters in the same column indicate significant differences based on Tukey’s HSD test (*p* ≤ 0.05). NS, *, **, and *** indicate no statistically significant difference and significant difference at *p* < 0.05, 0.01, and 0.001, respectively.

Seeds treated with 0, 10, 100, or 1000 mg∙L^-1^ of GA_3_ had 22.00% ± 5.03, 16.00% ± 5.16, 16.00% ± 5.89, and 57.00% ± 1.91 germination, respectively, at 15/6°C and 51.00% ± 9.14, 62.00% ± 7.39, 43.00% ± 11.24, and 94.00% ± 1.15 germination, respectively, at 25/15°C (Fig 5A and 5B). Regardless of the temperature regime, seeds treated with 1000 mg∙L^-1^ GA_3_ showed a decline in MGT values (Fig 5C and 5D). Furthermore, higher concentrations of GA_3_, 1000 mg∙L^-1^ of GA_3_ in particular, increased the germination velocity for TGI and showed a more positive effect at 25/15°C than at 15/6°C (Fig 5E and 5F).

## 4. Discussion

Baskin and Baskin [15] reported that the impermeability of intact seeds due to seed coat or pericarp is a characteristic feature of PY. If the water imbibition in seeds after 24 h increases by ≥ 20.00% of the initial seed mass under natural conditions, the seed is assumed to be classified as water-permeable [33]. In the present study, the seed mass increased by ≥ 32.35% ± 4.67 after 12 h of immersion in distilled water (Fig 1). Water absorption was not blocked by the impermeability of the seed coat in *C*. *takesimana* seeds, and the species did not exhibit PY.

At the time of seeds the dispersal the embryo was underdeveloped (Fig 2B) but the embryos grew notably during germination (Fig 2C). E:S ratio, which represents the relative size of embryos to the relative length of seeds, increased from 0.40 to 0.67 and showed a statistically significant difference compared with that at dispersal and before germination of seeds (Fig 2F). The length of seeds and embryos differed even within the same species. If the E:S ratio is ≤0.50, the embryo morphology is considered to be underdeveloped linearly [30]. Therefore, considering the E:S ratio of the *C*. *takesimana* seeds, it can be concluded that they exhibit MD. Generally, when seeds with an underdeveloped or undifferentiated embryo are dispersed from parent plants, MD or MPD is demonstrated, and the seeds may germinate or not within 30 days under suitable environmental conditions. These dormancy types require sufficient time for embryo development and radicle protrusion [18, 34]. If seed germination occurs within 30 days, it is determined as MD, and if seed germination does not occur within 30 days or pretreatments such as cold and/or warm stratification or phytohormones are required, it is determined as MPD [16, 35].

The germination of *C*. *takesimana* seeds at 25°C was significantly higher (37.00% ± 4.43) than that at other temperature regimes after seven weeks of incubation (Table 1). The seeds showed high germination of 27.00% ± 5.26 at 25/15°C and tended to germinate at the relatively high-temperature groups (≥ 20°C). Furthermore, most of the *C*. *takesimana* seeds germinated within two weeks under all temperature regimes, and there was no significant change until after seven weeks (Fig 3). Temperature is considered to be the most important environmental factor influencing the germination timing of seed dormancy [36]. The temperature required for optimal seed germination can be species-specific, and this is highly associated with the native habitat of plants. Hartmann et al. [37] reported that the range of critical germination temperature is 24 to 30°C for seeds of plants grown in temperate zones. Similarly, Koutsovoulou et al. [29] reported that approximately 23 species of the *Campanula* genus showed enhanced final germination at higher temperature regimes (≥ 20°C) compared with that at lower temperature regimes. In addition, germination velocity (28.58% ± 3.29 day^-1^) was significantly faster at 25°C than at other temperature regimes. Germination velocity, which is also known as seed efficiency, can provide more accurate quantification for seedling plans and/or forest restoration preparations [38, 39]. Generally, germination and germination velocity of seeds increase until a critical temperature is attained and then rapidly decrease when the temperature reaches the uppermost limit [40]. In this experiment with *C*. *takesimana* seeds, the relatively high-temperature regimes (25°C and 25/15°C) not only enhanced the initiation of seed germination but also increased the germination velocity. In summary, from the perspective of temperature and germination characteristics, 36.00% ± 4.32 of seeds germinated at 25°C within four weeks, even though they had underdeveloped embryos (Fig 3). Approximately 36.00% of the seeds had MD, and approximately 64.00% of the seeds had MPD; hence, dormancy-releasing treatment is required to improve germination.

In general, MPD in seeds can be broken by the application of successive temperature stratification (cold, 0–10°C; warm, ≥ 15°C; cold-warm; cold-warm-cold; and warm-cold), phytohormones, and chemical substance treatments. Stratification treatment does not only control seed dormancy by using the temperature required for seed germination but also by appropriately regulating germination timing [15, 41]. MPD is subdivided into nine classification based on the pretreatment required to overcome seed dormancy and the temperature required for growth up to the critical point before radicle protrusion [16]. The nine MPD levels are subdivided into two classification: simple and complex. Seeds with simple MPD require warm temperatures (≥ 15°C) for embryo development, whereas seed with complex MPD requires cold temperatures (approximately 0–10°C) for embryo development [15]. In the present study, seed germination significantly improved after cold stratification. Regardless of the levels of deep and intermediate PD, dormancy was broken even within a short period (e.g., four weeks) of cold stratification (Fig 4). In addition, when there was no seed germination for seven weeks at a temperature of 5°C and under a high temperature after cold stratification, the embryo grew to a critical point and germinated, indicating a simple level MPD. In stratification experiments, the season of dispersal from parent plant species in their native habitat of natural conditions is important. Seeds are commonly dispersed during the autumn season and can usually release dormancy through cold stratification. Reports showed that the germination of seeds dispersed in the autumn season notably improved dormancy release after pretreatment with cold stratification [11, 23, 42–46].

During the pretreatment of dormant seeds with cold stratification, hormonal changes, such as GA biosynthesis, occur by inducing the GA3ox1 and GA3ox2 genes [37, 47]. GAs not only promote germination by antagonizing abscisic acid (ABA) but also release dormant seeds [48]. Several studies have reported that endogenous phytohormones, especially GAs and ABA, regulate seed dormancy and germination [21, 46, 49–54]. In this study, GA_3_ pretreatment positively and effectively enhanced the germination characteristics of *C*. *takesimana* seeds. In particular, the germination and germination velocity of seeds significantly improved when pretreated with 1000 mg∙L^-1^ GA_3_ at an incubation temperature of 25/15°C than at 15/6°C. However, germination characteristics were positively affected when the concentration of GA_3_ increased at a low incubation temperature (15/6°C), suggesting that the species of *C*. *takesimana* seeds are sensitive to GA_3_. GA application promotes the production of enzymes that hydrolyze cell walls after seed maturation, thereby accelerating radicle protrusion by decomposing endosperm resistance. The balance between GAs and ABAs within seeds regulates the initiation, maintenance, and termination of seed dormancy. Therefore, the differences in the germination promotion of wild plants by exogenous GAs require further discussion and study.

## 5. Conclusion

Similar to other Campanulaceae families, the seeds of the native Korean *C*. *takesimana* species had underdeveloped embryos from their parent plants at the time of dispersal and showed MD that required embryo development before germination [13, 14]. Before radicle protrusion, the E:S ratio increased from 0.40 to 0.67, and the cumulative germination did not reach 36.00% within 30 d in the temperature regime groups. Therefore, the seeds are considered to have shallow levels of PD, and a short period (four weeks) of cold stratification is needed to release dormancy and enhance germination. Although cold stratification for a period of four weeks was used to produce seedlings, we observed that normal seedlings could be produced over a relatively shorter period by soaking the seeds in 1000 mg∙L^-1^ GA_3_ for 1 d under simulated spring temperatures (mean 25°C). In particular, GA_3_ can be a substitute for cold stratification. In summary, *C*. *takesimana* seeds have a non-deep level of PD, indicating that their dormancy can easily be broken by cold stratification and GA_3_. These results show that embryo growth occurs under high temperatures rather than under low temperatures, and the dormancy level is simple. In conclusion, *C*. *takesimana* seeds have a non-deep, simple MPD type that can be released by cold stratification and pretreatment with GA_3_ to enhance germination characteristics. The knowledge gained from this study would enable horticulturalists and seed ecologists to reduce the time required to produce *C*. *takesimana* seedlings and provide a useful reference for species conservation through mass propagation protocols.
